# Time Spent on Instagram and Body Image, Self-esteem, and Physical Comparison Among Young Adults in Spain: Observational Study

**DOI:** 10.2196/42207

**Published:** 2023-04-07

**Authors:** Isabel Alfonso-Fuertes, Miguel Angel Alvarez-Mon, Rafael Sanchez del Hoyo, Miguel A Ortega, Melchor Alvarez-Mon, Rosa M Molina-Ruiz

**Affiliations:** 1 Department of Psychology Comillas University Madrid Spain; 2 Department of Medicine and Medical Specialities Faculty of Medicine and Health Sciences University of Alcala Alcala de Henares Spain; 3 Ramón y Cajal Institute of Sanitary Research Madrid Spain; 4 Department of Psychiatry and Mental Health Hospital Universitario Infanta Leonor Madrid Spain; 5 Instituto de Investigación Sanitaria del Hospital Clínico San Carlos Madrid Spain; 6 Unidad de Apoyo Metodológico a la Investigación and Preventive Department Hospital Clínico San Carlos Madrid Spain; 7 Immune System Diseases-Rheumatology and Internal Medicine Service University Hospital Príncipe de Asturias Alcala de Henares (Madrid) Spain; 8 Department of Psychiatry and Mental Health Hospital Clínico San Carlos Madrid Spain

**Keywords:** Instagram, self-esteem, body image, physical comparison, young adults, social media, assessment, tool, questionnaire, satisfaction, physical appearance, usage

## Abstract

**Background:**

Instagram is a social media platform based on photos and videos that encourages interaction and comparison between users. Its growing popularity, especially among young people, has generated interest in the impact its use can have on users´ mental health, specifically on their self-esteem and degree of satisfaction with their own body image.

**Objective:**

We aimed to analyze the relationships between the use of Instagram, both the hours of daily use and the type of content viewed, and self-esteem, tendency to make physical comparisons, and satisfaction with body image.

**Methods:**

In this cross-sectional study, we recruited 585 participants aged between 18 years and 40 years. Individuals who were interested in participating but had a personal history of eating disorders or had previously been diagnosed with a psychiatric disorder were excluded. The assessment tools consisted of (1) a questionnaire that collected sociodemographic data and Instagram use variables and was created by the research team specifically for this study; (2) the self-esteem scale by Rosenberg; (3) Physical Appearance Comparison Scale-Revised (PACS-R); and (4) Body Shape Questionnaire (BSQ). The recruitment and evaluation processes were carried out in January 2021.

**Results:**

Of the participants, 234 (234/585, 40%) used Instagram less than 1 hour a day, 303 (303/585, 51.8%) used Instagram between 1 hour and 3 hours a day, and 48 participants (48/585, 8.2%) used it more than 3 hours per day. We found statistically significant differences (*P*<.05) between the 3 groups in the scores obtained on the self-esteem test by Rosenberg, PACS-R, and BSQ. Participants who spent more time on Instagram had higher levels of body dissatisfaction, greater comparisons of physical appearance, and lower self-esteem. Moreover, we analyzed the relationship between the score obtained on the different scales and the types of content viewed, with no differences between those who mainly viewed professional content and those who primarily consumed fashion and beauty or sport and nutrition content.

**Conclusions:**

The results of this study indicate that the use of Instagram is associated with poorer body image satisfaction and self-esteem, mediated by the tendency to compare physical appearance in relation to the daily duration of Instagram use.

## Introduction

Currently, there are approximately 3.5 billion active users on social media around the world [1]. The Internet has revolutionized the way people communicate, interact, and obtain information, no longer serving as resources only for younger users but instead having extended to other age groups [2]. Unlike traditional media, social media content is mostly generated by peers, so that users are simultaneously both receivers and sources of information [3].

Instagram is a social media platform based on photos and videos with a higher percentage of use among young adults between 18 years and 34 years of age, as well as women [4-7]. The platform allows users to upload photos that emphasize their physical attractiveness, which prior to posting can be edited using filters that are provided by the application [4]. As a consequence, there is an impact on stereotypes, beauty standards, and body image, especially among Instagram’s youngest users, who are the most susceptible [8]. This is due to the many opportunities that the platform offers to make comparisons between different users [6,9] caused by the wide variety of celebrities and peers who share idealized photographs of themselves [3,4].

Instagram’s growing popularity, the highly visual nature of published content, and the tendency of users to make comparisons, both in terms of appearance and other aspects of their lifestyles on display, have sparked great interest among researchers in investigating its relationship with user dissatisfaction, body image, and self-esteem. However, despite the proliferation of Instagram’s use, literature remains divided regarding the potential impact of social media, particularly regarding image-based platforms.

Moreover, most of the studies that have investigated the relationship between social media, self-esteem, and body image are mainly based on the use of Facebook [4,6-13]. Facebook has been the most widespread platform worldwide with the largest number of users [6,14]. However, the use of other social networks such as Instagram has increased dramatically in recent years [9,10,15]. These other platforms offer more diverse interaction and the ability to edit images, with a highly aesthetic and visual nature of their published content [6,9,12]. This new format can yield more relevant results in this field [3,5,10-12,16].

Holland and Tiggemann [16] concluded that the increase in the use of social media, such as Facebook, is linked to concerns about body image [14,15]. Moreover, the negative impact on the perception of body image is more pronounced in the more visual social media in which users basically post, edit, and comment on photographs [3-5,12,15]. This engagement, in particular, allows for the evaluation of both one's own physical attractiveness and that of others [4]. Recent studies have shown that greater photographic activity, such as the publication of photos and stories, along with the observations of photos shared by other users, is associated with greater vigilance over the body, internalization, the desire to achieve the ideal of thinness, and dissatisfaction with one’s own weight and body shape [6,10,15,17].

The aim of this study was to analyze the relationships between the use of Instagram, both the hours of daily use and the type of content viewed, and self-esteem, tendency to make physical comparisons, and satisfaction with body image. Finally, we hypothesized that participants with more hours of Instagram use as well as those who mainly follow fashion, beauty, sports, and nutrition accounts will be those with the lowest self-esteem, greatest tendency to make physical comparisons, and least satisfaction with their body image.

## Methods

### Design and Participants

In this cross-sectional, observational study, the target population consisted of Instagram users. The inclusion criteria were (1) age between 18 years and 40 years; (2) being an Instagram user; (3) Spanish nationality; and (4) favorable informed consent by the participant (sent electronically). Individuals who were interested in participating but who had a personal history of eating disorders or had previously been diagnosed with a psychiatric disorder were excluded. The study was mainly publicized through the Instagram account of one of the authors (RMMR), who is an influencer on Instagram and had more than 85,000 followers at the time of the study. In addition, the followers of this account and another of the authors (IAF) shared it with people potentially interested. All individuals interested in participating were given the survey to collect sociodemographic data and data related to Instagram use, as well as information related to the inclusion and exclusion criteria of the study. Thus, of the 680 individuals who filled out the survey, only 585 met the criteria to participate because the rest of participants did not answer the questionnaire completely or met an exclusion criterion. The recruitment process was carried out during the month of January 2021.

### Assessment Instruments and Variables Considered

We used the following measurement instruments: (1) questionnaire collecting sociodemographic data and Instagram use variables created by the research team specifically for this study; (2) self-esteem questionnaire by Rosenberg [18]; (3) Physical Appearance Comparison Scale-Revised (PACS-R); and (4) Body Shape Questionnaire (BSQ). The sociodemographic data questionnaire collected information regarding age, gender, employment status, marital status, number of children, education level completed, and socioeconomic status. Regarding the use of Instagram, we collected information concerning the number of hours of daily use, number of followers, number of accounts followed by the participant, and number of posts and stories published per week. Likewise, we classified the content visualized by the participant into 4 categories: (1) fashion and beauty (eg, content related to clothes, makeup, accessories, hairstyles, creams), (2) professional matters (posts related in some way to work), (3) sports and nutrition, and (4) others (any content that did not belong to the previous categories was classified as others).

The self-esteem test by Rosenberg [18] is among the most widely used scale for measuring global self-esteem. It evaluates the perception that the participant has of himself or herself. It consists of 10 items and a Likert-type response modality ranging from 1 (strongly disagree) to 4 (strongly agree). Based on the total score, 3 categories of self-esteem have been established: high (>30 points), medium (20-30 points), and low (<20 points). In its original version, it has a reliability of 0.92 and an internal consistency of 0.72 [18]. The reliabilities for Spanish samples are above 0.70 [19]. The PACS-R is a self-administered scale composed of 11 items that measures the frequency with which the person compares their physical appearance with others in various social situations [20]. The score on the scale varies between 0 and 44. It was adapted to Spanish by Senín Calderon and Rodriguez-Testal [21]. The BSQ is a self-administered questionnaire adapted to Spanish [22,23]. It is composed of 34 items (statements) that the participant scores between 1 (1=Never) and 6 (6=Always), so the total score of the test varies between a minimum score of 34 and a maximum score of 204. According to Cooper et al [23], 4 categories can be established from the total score based on the extent of body image concerns: no concern (score <80), slight concern (score between 81 and 110), moderate concern (score between 111 and 140), and extreme concern (score >141). It is important to note that this questionnaire is not a screening or diagnostic tool for eating disorders.

### Ethical Considerations

This study received approval from the University of Comillas Research Ethics Committee and was compliant with the research ethics principles of the Declaration of Helsinki (7th revision, 2013). Prior to their participation, all individuals gave their written consent. Nevertheless, we have de-identified the data to guarantee anonymity.

### Statistical Analysis

In the descriptive analysis of the data, the qualitative variables were summarized with the distribution of frequencies. Quantitative variables with a normal distribution are presented as the mean (SD) and, in the case of an asymmetric distribution, as the median (IQR). The homogeneity of the sample was observed by comparing the sociodemographic variables with the variable time of use of categorized Instagram. For the comparison of qualitative variables, the chi-square test was used. The comparisons of means between the 2 independent groups of the type of content viewed on Instagram (professional vs fashion and beauty plus sport and nutrition) were made using *t* tests or nonparametric Mann-Whitney *U* tests. The comparisons of means between the 3 independent groups of the duration of Instagram use (<1 hour, 1-3 hours, and >3 hours) were made with analyses of variance (ANOVAs) or nonparametric Kruskal-Wallis tests for asymmetric variables. A multiple linear regression model was used to explore the association between daily Instagram use in hours and each of the dependent variables (total BSQ, self-esteem scale, and total PACS-R). For each of the outcome variables, the gross and adjusted effects for sex, job, educational level, number of followers, and number of stories uploaded weekly were calculated. For all tests, a significance value of 5% was accepted. Data processing and analysis were performed using the statistical software SPSS v.26 (IBM Corp).

## Results

### Characterization of the Participants Based on the Daily Length of Instagram Use

Our sample was composed of 585 participants aged between 18 years and 40 years. As shown in Table 1, 234 of the 585 participants (40%) used Instagram less than 1 hour a day, 303 (51.8%) used Instagram between 1 hour and 3 hours a day, and 48 participants (8.2%) used Instagram more than 3 hours per day. Concerning the demographic characteristics of the participants, we found statistically significant differences between groups in gender (*P*=.02), employment status (*P*=.02), and educational level (*P*=.01). Women predominated over men in the 3 groups, but this predominance was greater in the group that reported using Instagram more than 3 hours per day (45/48, 94%) than in the group that reported using it less than 1 hour a day (191/234, 81.6%). Statistically significant differences were also found in the proportion of employed participants among the 3 groups. The percentage of employed workers in the group that reported using Instagram less than 1 hour a day (150/234, 64.1%) was higher than in the group that reported using it more than 3 hours a day (24/48, 50%). We also found statistically significant differences between the 3 groups in educational level. The percentage of participants with a college degree (170/234, 72.6%) was higher in the group with less daily use of Instagram, while the percentage of participants who had only completed the obligatory studies was higher (9/48, 19%) in the group that reported more than 3 hours of daily Instagram use.

Moreover, we studied the pattern of use and the user characteristics based on the duration of use (Table 2). We found statistically significant differences in the number of accounts followed and in the number of stories published per week between the 3 groups. The group of participants who reported using Instagram for at least 3 hours a day followed an average of 729 accounts, while the group of participants who reported using Instagram for less than 1 hour a day followed an average of 475 accounts. On the other hand, we found differences in the number of stories published among the different groups. In the group that made the most daily use of Instagram, the percentage of users who published more than 5 stories a week was higher (12/48, 25%) than that of the group who used Instagram less than 1 hour a day (20/234, 8.5%). However, we did not find statistically significant differences between the 3 groups in the number of followers or in the number of weekly posts published nor did we find differences in terms of the type of content visualized.

**Table 1:** Sociodemographic characteristics of the participants according to the duration of daily Instagram use.

Variables		Total sample (n=585)	Duration of daily Instagram use				*P* value	
			<1 hour (n=234)	1-3 hours (n=303)	>3 hours (n=48)			
Age (years), mean (SD)		26.8 (5.9)	27.1 (5.9)	26.8 (5.9)	25.1 (5.2)	.99		
Gender (female), n (%)		505 (86.3)	191 (81.6)	269 (88.8)	45 (93.8)	.02		
Currently employed (yes), n (%)		334 (57.1)	150 (64.1)	160 (52.8)	24 (50.0)	.02		
Children (yes), n (%)		55 (9.4)	20 (8.5)	29 (9.6)	6 (12.5)	.69		
**Educational level, n (%)**								.01
	High school/VT^a^	74 (12.6)	31 (13.2)	30 (9.9)	13 (27.1)			
	Middle school	90 (15.4)	33 (14.1)	48 (15.8)	9 (18.8)			
	College degree	421 (72.0)	170 (72.6)	225 (74.3)	26 (54.2)			
**Marital status, n (%)**								.71
	Single	262 (44.8)	107 (45.7)	136 (44.9)	19 (39.6)			
	In a relationship	249 (42.6)	98 (41.9)	127 (41.9)	24 (50.0)			
	Married	67 (11.5)	28 (12.0)	35 (11.6)	4 (8.3)			
	Divorced	7 (1.2)	1 (0.4)	5 (1.7)	1 (2.1)			
**Socioeconomic status, n (%)**								.16
	Low	36 (6.2)	9 (3.8)	20 (6.6)	7 (14.6)			
	Medium	352 (60.2)	137 (58.5)	186 (61.4)	29 (60.4)			
	Medium-high	187 (32.0)	84 (35.9)	92 (30.4)	11 (22.9)			
	High	10 (1.7)	4 (1.7)	5 (1.7)	1 (2.1)			

^a^VT: vocational training.

**Table 2:** Characterization of the participants’ use of Instagram based on the daily duration of use.

Variables			Total sample (n=585)		Duration of daily Instagram use							*P* value	
					<1 hour (n=234)		1-3 hours (n=303)		>3 hours (n=48)				
Number of followers, mean (range)			430.0 (218.0-700.5)		326.0 (169.0-615.5)		500.0 (250.0-760.0)		612.0 (329.0-1211.0)		.07		
Number of accounts followed, mean (range)			583.0 (349.5-825.5)		475.0 (300.0-749.0)		600.0 (357.0-866.0)		729.5 (522.0-990.0)		<.001		
Posts per week (≥1), n (%)			124 (21.2)		44 (18.8)		64 (21.1)		16 (33.3)		.08		
**Stories per week, n (%)**													<.001
	0	205 (35.0)		104 (44.4)		92 (30.4)		9 (18.8)					
	1-5	312 (53.3)		110 (47.0)		175 (57.8)		27 (56.3)					
	>5	68 (11.6)		20 (8.5)		36 (11.9)		12 (25.0)					
**Visualized content, n (%)**													.69
	Fashion and beauty	365 (62.4)		144 (61.5)		190 (62.7)		31 (64.4)					
	Professional	145 (24.8)		59 (25.2)		76 (25.1)		10 (20.8)					
	Sport and nutrition	44 (7.5)		19 (8.1)		19 (6.3)		6 (12.5)					
	Others^a^	31 (5.3)		12 (5.1)		18 (5.9)		1 (2.1)					

^a^Travel, landscapes, gastronomy, humor, and motor.

Finally, we analyzed the relationship between the time spent on Instagram and satisfaction with body image, self-esteem, and the tendency to compare oneself with others (Table 3). After adjusting for the variables that we identified as different between the 3 groups (gender, employment, educational level, number of accounts followed, and number of stories published per week), we found that those participants who used Instagram between 1 hour and 3 hours or more than 3 hours had higher scores on the BSQ: that is, the greater the daily use of Instagram, the greater dissatisfaction with body image. Likewise, we found that those participants with more hours of daily Instagram use had higher scores on the PACS-R. Finally, we also found a relationship between the use of Instagram and self-esteem. Those participants with more hours of Instagram use tended to have lower self-esteem. In fact, we found that participants who reported using Instagram for more than 3 hours per day scored significantly lower than those participants who used Instagram for less than 1 hour per day.

**Table 3:** Comparison between body dissatisfaction, self-esteem, and physical appearance comparison with length of daily use of Instagram adjusted by gender, employment status, educational level, number of accounts followed, and number of stories published weekly.

Questionnaire	Daily duration of Instagram use	*β* (95% CI)^a^	*P* value
**Body dissatisfaction (BSQ^b^)**			
	Between 1 hour and 3 hours	7.75 (1.08 to 14.42)	.02
	More than 3 hours	28.08 (15.81 to 40.35)	<.001
**Self-esteem**			
	Between 1 hour and 3 hours	–0.03 (–1.11 to 1.04)	.95
	More than 3 hours	–2.84 (–4.82 to –0.86)	.005
**PACS-R^c^**			
	Between 1 hour and 3 hours	2.22 (0.41 to 4.40)	.046
	More than 3 hours	5.91 (1.90 to 9.92)	.004

^a^Reference category: <1 hour.

^b^BSQ: Body Shape Questionnaire.

^c^PACS-R: Physical Appearance Comparison Scale-Revised.

### Characterization of the Participants Based on the Type of Content Visualized on Instagram

Based on the content viewed on Instagram, we classified the participants into 2 groups: those who mainly viewed professional content and those who viewed content related to fashion and beauty plus sports and nutrition (Table 4). Interestingly, we found statistically significant differences in several variables between groups. The mean age was significantly older in the group that preferentially viewed professional content than in the group that viewed fashion and beauty. Women clearly predominated in both groups but were more prominent in the group that viewed professional content. Participants with children were a minority in both groups, but in the group that predominantly viewed professional content, they outnumbered the other group by 3 times.

Regarding the type of user and pattern of use of the platform, we found that users who preferably viewed beauty and fashion content had significantly more followers than users who viewed basically professional content (Table 5). Likewise, they also followed more accounts. However, there were no significant differences between the 2 groups in the number of posts and stories published weekly.

Moreover, we analyzed the relationship between the score on the different scales (self-esteem test by Rosenberg, PACS-R, and BSQ) and the type of content viewed, without finding differences in the scores obtained between those participants who basically viewed professional content and those who primarily viewed beauty and fashion content.

**Table 4:** Sociodemographic characteristics of the participants according to the type of content viewed on Instagram.

Variables			Professional (n=145)		Fashion and beauty plus sports and nutrition (n=409)		*P* value	
Age (years), mean (SD)			29.42 (6.33)		25.61 (5.37)		<.001	
Gender (female), n (%)			136 (93.8)		347 (84.8)		.006	
Currently employed (yes), n (%)			88 (60.7)		226 (55.3)		.26	
Children (yes), n (%)			30 (20.7)		25 (6.1)		<.001	
**Educational level, n (%)**								.03
	High school/VT^a^	19 (13.1)		49 (12.0)				
	Middle school	13 (9.0)		74 (18.1)				
	College degree	113 (77.9)		286 (69.9)				
**Family status, n (%)**								<.001
	Single	48 (33.1)		199 (48.7)				
	In a relationship	62 (42.8)		175 (42.8)				
	Married	32 (22.1)		31 (7.6)				
	Divorced	3 (2.1)		4 (1.0)				
**Socioeconomic status, n (%)**								.03
	Low	12 (8.3)		24 (5.9)				
	Medium	99 (68.3)		235 (57.5)				
	Medium-high	32 (22.1)		142 (34.7)				
	High	2 (1.4)		8 (2.0)				

^a^VT: vocational training.

**Table 5:** Characterization of the participants’ use of Instagram based on the type of content viewed on Instagram.

Variables		Professional (n=145)	Fashion and beauty plus sport and nutrition (n=409)	*P* value
Number of followers, mean (range)		250.0 (113.5-500.0)	500.0 (287.0-758.0)	<.001
Number of accounts followed, mean (range)		450.0 (250.0-800.0)	600.0 (385.0-831.0)	.02
Posts per week (≥1), n (%)		37 (25.5)	79 (19.3)	.12
**Stories per week, n (%)**				.27
	0	58 (40.0)	134 (32.8)	
	1-5	71 (49.0)	230 (56.2)	
	>5	16 (11.0)	45 (11.0)	

## Discussion

### Main Findings

This study raises awareness of the impact of Instagram use on self-esteem, body dissatisfaction, and tendency for self-comparison. In the first place, the results of this study show that the users who spent more time on Instagram (especially the group that used the platform more than 3 hours a day) were those who had higher levels of body dissatisfaction, greater comparisons of physical appearance, and lower self-esteem. However, the type of content viewed did not affect the score on the questionnaires.

### Social Comparison, Gender, and Time Spent on Instagram

Instagram is used to a greater extent by women, who are more likely to use the platform to make self-comparisons than men [24]. Moreover, women tend to spend more time observing people of the same sex than men, so they may be more vulnerable to the evaluations that other users make of their posts or publications [12,25]. In addition, they have traditionally been more exposed to beauty and image advertisements that shape the conceptualization of ideal female beauty and create stereotypes that are increasingly unrealistic, promoting female body dissatisfaction [9,26]. Mclean et al [27] found that women who regularly shared photos of themselves on social media also showed greater internalization of thinness ideals and dissatisfaction with their body image.

Moreover, in our study, women engaged more with Instagram than men, and further, they engaged more intensely with the platform. However, the gender factor was controlled in this study when assessing whether the length of time spent using the platform determined variation within the clinical scales. In fact, the most relevant finding was the relationship between the time users spent on Instagram and the score obtained on the scales, which suggests that participants with higher levels of body dissatisfaction, a greater comparison of physical appearance, and lower self-esteem were those who spent more time using Instagram. Indeed, on average, participants who used the platform for more than 3 hours a day scored 28.08 points higher on the BSQ, 2.84 points less on the self-esteem scale, and 2.22 points higher on the scale measuring one’s tendency to compare physical appearance. These results are relevant from a clinical point of view. A previous study in which 3825 adolescents were followed for 4 years found the longer the time spent using social media, the lower self-esteem and emotional well-being they showed [28]. This association does not seem to have anything to do with the screen time, since other screen uses, such as time spent playing video games, were not associated with lower self-esteem or greater emotional distress [28]. Based on the upward social comparisons, it might be possible that repeated exposure to idealized images lowers self-esteem and worsens mental well-being, but this is not the case with other, different platforms such as those related to video games. Furthermore, heavier users of Instagram with lower self-esteem appear to be more negatively affected by their time spent on social media, potentially by how the content viewed affects them. In addition, it has been demonstrated that mood level affects the content selection [29].

Furthermore, our results are consistent with research findings that explored this same relationship with other visual platforms such as Facebook, stating that greater use of the platform was related to higher levels of body dissatisfaction and body vigilance among pre-adolescent girls [30] and university students [14,31]. Additionally, studies carried out by Tazghini and Siedlecki [32] and Faraon and Kaipainen [33] showed that greater Facebook use was associated with worse psychological well-being and self-esteem. In relation to Instagram, the study by Sherlock and Wagstaff [11] found that time spent on Instagram was associated with low self-esteem, general and physical appearance anxiety, and body dissatisfaction among women. In addition, the work by Fardouly and Vartanian [14], along with that of Feltman and Szymansky [31], showed that regularly comparing physical appearance with others mediated the relationship between the use of social networks and changes in body dissatisfaction. Additionally, time spent exposed to the content of others seemed to predict or explain, in part, users’ low self-esteem. This was reflected in the study by Vogel et al [34] that concluded that those who maintained greater exposure to Facebook tended to show worse self-esteem.

This phenomenon can be explained by means of Festinger's Theory of Social Comparison, which postulates that, when people lack an objective point of reference to determine their own progress and social position, they often evaluate themselves through comparisons with others they consider relevant [4,9]. For this reason, a growing body of research argues that social comparison processes mediate the association between social media use and body image concerns [6,15]. On Instagram, there are greater opportunities to make comparisons, whereby individual users tend to perceive themselves more negatively [4,6,14,35]. Possible discrepancies that users may find between their bodies and ideals of beauty can impact perceptions of body dissatisfaction, as well as generate low self-esteem [36]. Additionally, the influence of these observed behaviors increases when they are perceived by individuals they consider outstanding and relevant, as explained in Bandura's Theory of Social Cognition [10]. Thus, the ideal of thinness shown in population imagery on social media networks can promote changes in dietary behavior and body dissatisfaction [37]. Specifically, on Instagram, peers become sources of vicarious learning, meaning that learning from behavioral observation or from factual information allows users to learn about editing techniques, evaluation standards among peers, feedback expectations, and evaluation as evidenced by the number of followers users have or the likes needed to validate their physical beauty [37-39].

### Relevant Factors Beyond the Time of Use

There is controversy as to what other factors, in addition to the length of use, may be related to satisfaction with body image, self-esteem, and the tendency to make physical comparisons. According to our results, the type of content viewed does not seem to be an influential factor. Moreover, previous studies have highlighted that an active disposition in the use of social media (ie, publishing photos, posts, or videos) can be protective, while a passive attitude in which the user is barely a content consumer has been associated with worse general health indicators [40,41]. Interestingly, something similar has also been described in other areas. For example, people who spend many hours watching television have worse health measures, while those who cultivate hobbies that require some type of effort have greater life satisfaction, quality of life, and cognitive performance [42,43]. On the other hand, previous research suggests positive consequences of active social networking that can lead to enhanced social capital and a sense of belonging [44-46]. On the other hand, passive use often leads to more harmful effects such as low self-esteem and reduced satisfaction with life [44,45,47]. However, regarding incorporation of the Passive and Active Use Measure, this might be difficult to accomplish since these categories are not mutually exclusive. Essentially, users can be both active and passive—even during the same online session. Thus, it is impossible to divide respondents into mutually exclusive groups. Moreover, for those with an active role, we did not differentiate among those who are more involved in selfie images or those who are more engaged in other kinds of content (ie, knowledge, professional, travel).

Previous research postulates that exposure to appearance-based images in the media negatively affects women's body image and self-esteem [3,11,48,49] . These results are surprising and may also have to do with how health campaigns in fashion and image are evolving on social networks, such as those known as “body positivity,” a growing social media trend that seeks to challenge dominant societal appearance ideals and promote acceptance and appreciation of all bodies and appearances, as well as self-compassion [5,35,50,51] . These movements that have emerged on social networks in recent years have especially grown during the pandemic, which might have influenced our data.

However, regarding the type of content consumed, we found that people who follow professional content are significantly older than those who consume fashion and beauty content. Likewise, a greater percentage of people with higher education was observed among those who followed work and interest accounts (113/145, 77.9%) compared with those who followed fashion and beauty accounts (286/409, 69.9%), in addition to a lower percentage of single users among those who followed work and interest accounts (48/145, 33.1%) compared with those who followed fashion and beauty accounts (199/409, 48.7%). Additionally, there was a higher percentage of users with children among those who followed professional accounts (30/145, 20.7%) than those who followed fashion and beauty accounts (25/409, 6.1%). Last, those who followed fashion and beauty accounts had more followers than those who followed professional accounts. All these sociodemographic variables could also explain the heterogeneity found in our sample in terms of Instagram use.

### Strengths and Limitations

Our study is the first to analyze the relationship between the use of Instagram in terms of time spent and type of content viewed with self-esteem, satisfaction with body image, and the tendency to make physical comparisons. The participant recruitment methodology using Instagram is novel, among other reasons, because few professionals with clinical practice and research activity are also successful influencers on Instagram. However, it seems to us an advantage since it allows us to study a homogeneous and representative Spanish sample. However, our study has some limitations that may affect the interpretation of the results. First, given that our study was cross-sectional, it was not possible to determine whether it was the time users spent on Instagram that led to greater body dissatisfaction and worse self-esteem or whether it was users’ concerns with their appearance prior to using Instagram that has resulted in greater time spent on the platform. Second, we did not carry out a clinical characterization nor did we carry out a psychological profile of the participants, which is relevant because, in previous studies, certain personality traits were predisposed to internalize ideals transmitted over media [52]. Third, it is worth mentioning that many of the items included in the BSQ scale were developed for people with eating disorders as well as validated in women. This could therefore lead to bias in the results in male participants. Finally, the population studied in this work had the same nationality, belonged to the same age group, and was interested in mental health (since they found out about this study by following this type of content on social networks), so future studies will determine if the results found in this work can be extrapolated to other populations.

### Conclusions

The results of this study indicate that the use of Instagram is associated with poorer satisfaction with body image and self-esteem, mediated by the tendency to compare physical appearance in relation to the duration of use. Although most studies have focused on women, due to the great pressure that the media and social media have exerted on women’s body image, this study shows that, even though women present with higher levels of body image dissatisfaction and lower self-esteem, Instagram has potential negative effects on both genders. Together, there are still many open questions on how social media affects people’s views on themselves and others, and future research may include more specific analysis.

## Abbreviations

ANOVA: analysis of variance
BSQ: Body Shape Questionnaire
PACS-R: Physical Appearance Comparison Scale-Revised

## Data Availability

The data sets generated during and/or analyzed during the current study are available from the corresponding author on reasonable request.

## References

[1] McCrory A, Best P, Maddock A (2020). The relationship between highly visual social media and young people’s mental health: A scoping review. Children and Youth Services Review.

[2] Pittman M, Reich B (2016). Social media and loneliness: Why an Instagram picture may be worth more than a thousand Twitter words. Computers in Human Behavior.

[3] Tiggemann M, Hayden S, Brown Z, Veldhuis J (2018). The effect of Instagram "likes" on women's social comparison and body dissatisfaction. Body Image.

[4] Senín-Calderón C, Santos-Morocho J, Rodríguez-Testal JF (2020). Validation of a Spanish Version of the Physical Appearance Comparison Scales. Int J Environ Res Public Health.

[5] Slater A, Varsani N, Diedrichs PC (2017). #fitspo or #loveyourself? The impact of fitspiration and self-compassion Instagram images on women's body image, self-compassion, and mood. Body Image.

[6] Engeln R, Loach R, Imundo MN, Zola A (2020). Compared to Facebook, Instagram use causes more appearance comparison and lower body satisfaction in college women. Body Image.

[7] Karim F, Oyewande AA, Abdalla LF, Chaudhry Ehsanullah R, Khan S (2020). Social media use and its connection to mental health: a systematic review. Cureus.

[8] Verrastro V, Fontanesi L, Liga F, Cuzzocrea F, Gugliandolo MC (2020). Fear the Instagram: beauty stereotypes, body image and Instagram use in a sample of male and female adolescents. QWERTY.

[9] Couture Bue AC, Harrison K (2020). Visual and cognitive processing of thin-ideal Instagram images containing idealized or disclaimer comments. Body Image.

[10] Charmaraman L, Sode O, Bickham D, Moreno MA, Hoopes AJ (2020). Adolescent mental health challenges in the digital world. Technology and Adolescent Health: In Schools and Beyond.

[11] Sherlock M, Wagstaff DL (2019). Exploring the relationship between frequency of Instagram use, exposure to idealized images, and psychological well-being in women. Psychology of Popular Media Culture.

[12] Couture Bue AC (2020). The looking glass selfie: Instagram use frequency predicts visual attention to high-anxiety body regions in young women. Computers in Human Behavior.

[13] Stapleton P, Luiz G, Chatwin H (2017). Generation validation: the role of social comparison in use of Instagram among emerging adults. Cyberpsychol Behav Soc Netw.

[14] Fardouly J, Vartanian LR (2015). Negative comparisons about one's appearance mediate the relationship between Facebook usage and body image concerns. Body Image.

[15] Fardouly J, Vartanian L (2016). Social media and body image concerns: current research and future directions. Current Opinion in Psychology.

[16] Holland G, Tiggemann M (2016). A systematic review of the impact of the use of social networking sites on body image and disordered eating outcomes. Body Image.

[17] Kim JW, Chock TM (2015). Body image 2.0: Associations between social grooming on Facebook and body image concerns. Computers in Human Behavior.

[18] Rosenberg M (1965). Society and the Adolescent Self-Image.

[19] Vázquez Morejón AJ, Jiménez García-Bóveda R, Vázquez-Morejón Jiménez R (2004). Escala de autoestima de Rosenberg: Fiabilidad y validez en población clínica española [The Rosenberg Self-esteem Scale: Reliability and validity in clinical samples of the Spanish population]. Apuntes de Psicología.

[20] Schaefer LM, Thompson JK (2014). The development and validation of the Physical Appearance Comparison Scale-Revised (PACS-R). Eat Behav.

[21] Senín-Calderón C, Rodriguez-Testal JF (2019). Validation of the physical appearance comparison scale-revised (PACS-R) in a Spanish population. European Psychiatry.

[22] Raich RM, Mora M, Soler A, Avila C, Clos I, Zapater L (1996). Adaptación de un instrumento de evaluación de la insatisfacción corporal [Adaptation of a body dissatisfaction assessment instrument]. Clínica y Salud.

[23] Cooper PJ, Taylor MJ, Cooper Z, Fairbum CG (1987). The development and validation of the body shape questionnaire. International Journal of Eating Disorders.

[24] Cristinzio C, N'Diaye K, Seeck M, Vuilleumier P, Sander D (2010). Integration of gaze direction and facial expression in patients with unilateral amygdala damage. Brain.

[25] Sabik NJ, Falat J, Magagnos J (2019). When self-worth depends on social media feedback: associations with psychological well-being. Sex Roles.

[26] Rodin J, Silberstein L, Striegel-Moore R (1984). Women and weight: a normative discontent. Nebr Symp Motiv.

[27] McLean SA, Paxton SJ, Wertheim EH, Masters J (2015). Photoshopping the selfie: Self photo editing and photo investment are associated with body dissatisfaction in adolescent girls. Int J Eat Disord.

[28] Boers E, Afzali MH, Newton N, Conrod P (2019). Association of screen time and depression in adolescence. JAMA Pediatr.

[29] Carpentier FRD, Brown JD, Bertocci M, Silk JS, Forbes EE, Dahl RE (2008). Sad kids, sad media? Applying mood management theory to depressed adolescents' use of media. Media Psychol.

[30] Tiggemann M, Slater A (2013). NetTweens. The Journal of Early Adolescence.

[31] Feltman C, Szymanski DM (2017). Instagram use and self-objectification: the roles of internalization, comparison, appearance commentary, and feminism. Sex Roles.

[32] Tazghini S, Siedlecki KL (2013). A mixed method approach to examining Facebook use and its relationship to self-esteem. Computers in Human Behavior.

[33] Faraon M, Kaipainen M (2015). Much more to it: The relation between Facebook usage and self-esteem.

[34] Vogel E, Rose J, Roberts L, Eckles K (2014). Social comparison, social media, and self-esteem. Psychology of Popular Media Culture.

[35] Brown Z, Tiggemann M (2020). A picture is worth a thousand words: The effect of viewing celebrity Instagram images with disclaimer and body positive captions on women's body image. Body Image.

[36] Thompson JK (1990). Body image disturbance: Assessment and treatment.

[37] Chua THH, Chang L (2016). Follow me and like my beautiful selfies: Singapore teenage girls’ engagement in self-presentation and peer comparison on social media. Computers in Human Behavior.

[38] Meier EP, Gray J (2014). Facebook photo activity associated with body image disturbance in adolescent girls. Cyberpsychol Behav Soc Netw.

[39] Krayer A, Ingledew DK, Iphofen R (2008). Social comparison and body image in adolescence: a grounded theory approach. Health Educ Res.

[40] Trifiro B (2018). Instagram Use and Its Effect on Well-Being and Self-Esteem. Bryant University Master Theses.

[41] Bodroža B, Obradović V, Ivanović S (2022). Active and passive selfie-related behaviors: Implications for body image, self-esteem and mental health. Cyberpsychology: Journal of Psychosocial Research on Cyberspace.

[42] Verghese J, Lipton RB, Katz MJ, Hall CB, Derby CA, Kuslansky G, Ambrose AF, Sliwinski M, Buschke H (2003). Leisure activities and the risk of dementia in the elderly. N Engl J Med.

[43] Basterra-Gortari FJ, Bes-Rastrollo M, Gea A, Núñez-Córdoba JM, Toledo E, Martínez-González MÁ (2014). Television viewing, computer use, time driving and all-cause mortality: the SUN cohort. J Am Heart Assoc.

[44] Krasnova H, Wenninger H, Widjaja T, Buxmann P (2013). Envy on Facebook: a hidden threat to users' life satisfaction?. Wirtschaftsinformatik Proceedings 2013.

[45] Verduyn P, Ybarra O, Résibois M, Jonides J, Kross E (2017). Do social network sites enhance or undermine subjective well-being? A critical review. Social Issues and Policy Review.

[46] Ellison NB, Steinfield C, Lampe C (2007). The benefits of Facebook “friends:” social capital and college students’ use of online social network sites. Journal of Computer-Mediated Communication.

[47] de Vries L, Peluso AM, Romani S, Leeflang PS, Marcati A (2017). Explaining consumer brand-related activities on social media: An investigation of the different roles of self-expression and socializing motivations. Computers in Human Behavior.

[48] Hogue JV, Mills JS (2019). The effects of active social media engagement with peers on body image in young women. Body Image.

[49] Groesz LM, Levine MP, Murnen SK (2002). The effect of experimental presentation of thin media images on body satisfaction: a meta-analytic review. Int J Eat Disord.

[50] Tiggemann M, Anderberg I, Brown Z (2020). #Loveyourbody: The effect of body positive Instagram captions on women's body image. Body Image.

[51] Retallack H, Ringrose J, Lawrence E, Coffey J, Budgeon S, Cahill H (2016). “Fuck Your Body Image”: Teen Girls’ Twitter and Instagram Feminism in and Around School. Learning Bodies. Perspectives on Children and Young People, vol 2.

[52] Yamamiya Y, Cash TF, Melnyk SE, Posavac HD, Posavac SS (2005). Women's exposure to thin-and-beautiful media images: body image effects of media-ideal internalization and impact-reduction interventions. Body Image.

